# *Lavandula angustifolia* and *Echium amoenum* as emerging adjuncts to cognitive behavioral therapy for major depressive disorder: mechanistic insights into neurotrophic, monoaminergic, and anti-inflammatory pathways

**DOI:** 10.3389/fpsyt.2026.1776230

**Published:** 2026-07-24

**Authors:** Yuhan Song, Jinlang Yang, Feng Xiao

**Affiliations:** 1 College of Arts and Sciences, Cornell University, Ithaca NY, United States; 2 Faculty of Life Sciences, University College London, London, United Kingdom; 3 Student Affairs Department, Anhui Jianzhu University, Hefei, Anhui, China

**Keywords:** behavioral therapy, brain, depression, *Echium amoenum*, *Lavandula angustifolia*

## Abstract

Major depressive disorder (MDD) represents a complex psychiatric disorder characterized by the interplay of psychological, neurobiological, and inflammatory factors. Cognitive behavioral therapy (CBT) is recognized as the primary non-pharmacological intervention; nevertheless, a significant number of individual’s experience either partial or delayed therapeutic outcomes, thereby underscoring the necessity for safe adjunctive approaches. *Lavandula angustifolia* (lavender) and *Echium amoenum* have been identified as promising phytotherapeutic options exhibiting antidepressant and anxiolytic effects, bolstered by an expanding body of both preclinical and clinical research. This review aims to synthesize contemporary insights regarding the potential efficacy of these herbal remedies as adjuncts to CBT in the context of MDD, emphasizing the relevant molecular and neurobiological pathways involved. Both *L. angustifolia* and *E. amoenum* have been shown to influence monoaminergic neurotransmission, specifically the serotonergic, dopaminergic, and noradrenergic systems, which are pivotal to the regulation of mood and cognitive function. These botanical species demonstrate neurotrophic properties through the enhancement of brain-derived neurotrophic factor levels and associated signaling pathways, thus facilitating synaptic plasticity and neurogenesis. Their anti-inflammatory and antioxidant properties, achieved by the inhibition of pro-inflammatory cytokines and oxidative stress. In summary, the incorporation of *L. angustifolia* and *E. amoenum* into CBT signifies an innovative, multimodal strategy that resonates with individualized and preventive mental health paradigms, necessitating further exploration into the underlying mechanisms and clinical implications.

## 1 Introduction

Major depressive disorder (MDD) constitutes a predominant contributor to global disability, impacting hundreds of millions of individuals across various demographic age categories and cultural backgrounds. This psychiatric condition is delineated by a sustained state of diminished mood, anhedonia, cognitive impairments, and considerable psychosocial dysfunction, which collectively heighten the risk of suicide and impose significant economic and healthcare challenges on a global scale. Epidemiological data indicate that the worldwide prevalence of depressive disorders is on an upward trajectory, with a disproportionate effect observed among females and younger populations, thereby establishing it as one of the most commonly diagnosed psychiatric conditions in clinical settings. Although specific statistics may differ regionally, the extensive repercussions of depression on quality of life and daily functioning are indisputable, necessitating continued endeavors to refine treatment methodologies beyond conventional monotherapy approaches (1–3). Pathophysiologically, MDD is increasingly conceptualized as a complex, multifactorial condition characterized by interrelated neurobiological mechanisms. Traditional monoaminergic theories emphasize the dysregulation of neurotransmitters, including serotonin, norepinephrine, and dopamine; however, these neurotransmitter-focused explanations fail to comprehensively account for the observed clinical heterogeneity and resistance to treatment. Modern integrative models encompass a range of neurobiological deficits, including diminished levels of brain-derived neurotrophic factor, hyperactivity of the hypothalamic-pituitary-adrenal (HPA) axis, neuroinflammatory processes, oxidative stress, as well as disturbances in neuroplasticity, immune responses, and stress reactivity. These converging mechanisms indicate that focusing solely on individual pharmacological targets may prove inadequate for addressing the entire array of biological disruptions linked to depressive symptomatology (4–6).

Despite the existence of an array of first-line antidepressant pharmacotherapies and rigorously substantiated psychotherapeutic interventions such as cognitive behavioral therapy (CBT), substantial deficiencies endure. Antidepressants, encompassing selective serotonin reuptake inhibitors and various other classifications, frequently necessitate an extended duration to elicit clinical efficacy, reveal inadequate response rates, and possess a potential for adverse effects that lead to premature cessation and suboptimal adherence. Similarly, although psychotherapy exhibits considerable effectiveness in a substantial number of patients, its advantages may be limited by accessibility constraints, extended treatment timelines, and variability in the availability and caliber of therapists. Furthermore, remission rates remain relatively modest, with a considerable fraction of individuals enduring residual symptoms notwithstanding the implementation of combined pharmacotherapy and psychotherapy (7–9). These deficiencies highlight the imperative for supplementary or integrative treatment methodologies that can optimize therapeutic outcomes and tailor care to individual patient needs.

In light of these inadequately addressed requirements, there has been an increasing scholarly interest in herbal and phytotherapeutic adjuncts that possess the ability to interact with multiple pathophysiological pathways associated with depression. Medicinal flora, historically employed within diverse complementary and integrative medicine paradigms, harbor bioactive compounds endowed with neurochemical, anti-inflammatory, antioxidant, and neuromodulatory attributes. For instance, *Lavandula angustifolia* (true lavender) has exhibited both anxiolytic and mood-regulating properties in preclinical and clinical investigations, potentially via the modulation of GABAergic and monoaminergic systems alongside effects on central neural circuits (10). Similarly, *Echium amoenum* encompasses fatty acid profiles and antioxidant components that may affect stress-related pathways and inflammatory responses, thereby indicating its potential as a mood adjunct (11, 12).

Significantly, although numerous systematic reviews and meta-analyses have assessed the efficacy of herbal medicines in the treatment of depression comprehensively, there exists a paucity of research that specifically investigates the roles of *Lavandula angustifolia* and *Echium amoenum* as adjunctive agents in conjunction with CBT, or that explores their mechanistic effects on neurotrophic, monoaminergic, and anti-inflammatory mediators within a cohesive mechanistic framework. Current reviews predominantly concentrate on either the efficacy of herbal interventions in isolation or general outcomes of phytotherapy, failing to integrate mechanistic evidence with clinical relevance, and neglecting to contextualize these herbs within the adjunctive framework of CBT. Consequently, this review is warranted to address this particular deficiency by rigorously synthesizing both mechanistic insights and emerging clinical evidence that advocate for Lavandula and Echium as complementary agents in the treatment of MDD. Such integration is imperative to advance translational research and to facilitate clinical decision-making towards more precise and multimodal interventions for depression.

## 2 Literature search strategy

This narrative review was conducted through a comprehensive literature search in PubMed, Scopus, and Web of Science databases. Relevant studies published up to November 2025 were identified using combinations of the following keywords: “*Lavandula angustifolia*,” “*Echium amoenum*,” “major depressive disorder,” “cognitive behavioral therapy,” “neuroinflammation,” “BDNF,” and “monoamine.” Only human clinical studies (randomized controlled trials, double-blind trials, and controlled clinical studies) were included in this review. Articles were selected based on their relevance to the neurobiological mechanisms and therapeutic effects of the mentioned herbal compounds in depression. No animal or *in vitro* studies were included in this review. Given the narrative nature of this review, no formal systematic inclusion or exclusion criteria were applied; however, efforts were made to ensure a balanced and representative overview of the existing clinical literature. Data from included clinical studies were extracted in a structured manner, including study design, sample size, type of intervention and standardized herbal preparation (e.g., aqueous extract, tincture, or standardized essential oil such as Silexan), dosage, duration of intervention, outcome measures, statistical significance (p-values where available), and study limitations. For clarity, all included clinical trials were systematically summarized in a structured format to facilitate comparison of efficacy, safety, and methodological quality across studies.

## 3 CBT in MDD

### 3.1 Core principles and clinical efficacy of CBT

**Figure 1:**
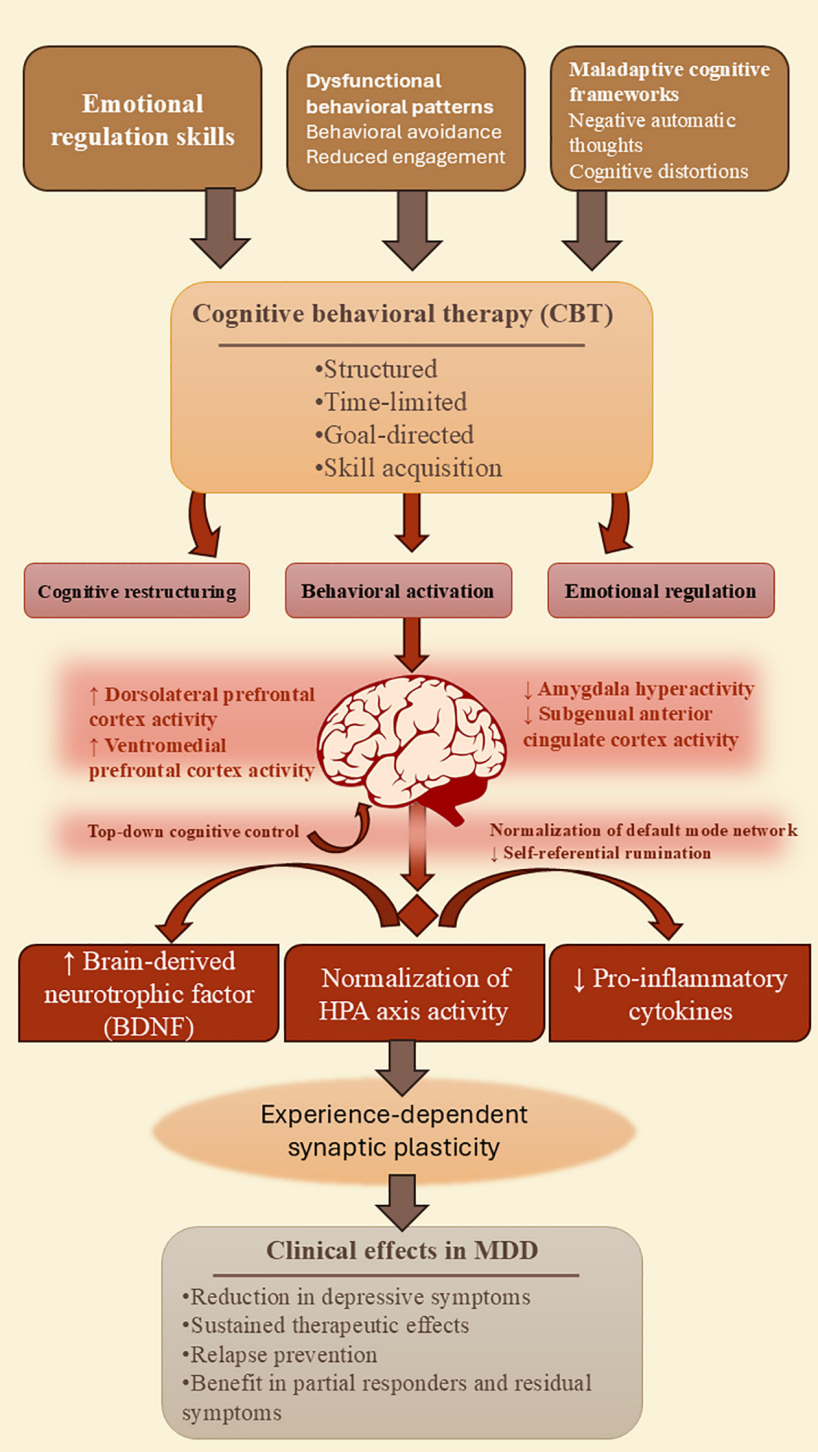
Cognitive behavioral therapy (CBT) in major depressive disorder exerts therapeutic effects through integrated psychological, neural, and biological mechanisms. By restructuring maladaptive cognitions and behaviors, CBT enhances top-down prefrontal control over limbic regions, normalizes resting-state network connectivity, promotes neurotrophic signaling, regulates stress-related neuroendocrine activity, and modulates inflammatory processes. These converging pathways contribute to symptom reduction and relapse prevention, while individual variability and incomplete biological normalization highlight intrinsic limitations of CBT alone.

CBT is recognized as one of the most rigorously validated psychotherapeutic modalities for the treatment of MDD (**Figure 1**). This intervention is predicated on the theoretical premise that maladaptive cognitive frameworks and dysfunctional behavioral patterns significantly contribute to both the onset and maintenance of depressive symptomatology. CBT utilizes a structured, temporally finite, and goal-directed approach to identify and amend negative automatic cognitions, cognitive distortions, and behavioral avoidance strategies. Through the promotion of active skill acquisition, CBT empowers patients to effectively manage emotional responses and stressors even after the conclusion of formal therapeutic intervention (13, 14). From a clinical perspective, CBT exhibits efficacy that is comparable to that of antidepressant pharmacotherapy across the spectrum of depressive disorders, encompassing both mild and severe presentations, whether administered as a singular therapeutic modality or in conjunction with pharmacological treatment (15). Comprehensive meta-analyses and randomized controlled trials (RCTs) have consistently demonstrated that CBT produces sustained therapeutic outcomes, particularly in the context of relapse prevention following the cessation of treatment (16, 17). Notably, CBT has been found to confer specific advantages for individuals exhibiting partial treatment response, residual symptomatology, or intolerance to pharmacological interventions.

### 3.2 Neurobiological correlates of CBT

Neuroimaging studies have provided compelling evidence that CBT induces measurable changes in brain function and connectivity (18). Functional MRI and PET investigations demonstrate that CBT modulates activity within cortico-limbic circuits central to emotional regulation (19). Specifically, CBT is associated with increased activation of the dorsolateral and ventromedial prefrontal cortex, alongside reduced hyperactivity in limbic regions such as the amygdala and subgenual anterior cingulate cortex (20). These findings suggest that CBT strengthens top-down cognitive control over emotion-generating neural systems (20). Furthermore, empirical evidence indicates that CBT has the capacity to normalize resting-state functional connectivity within the default mode network, a neural system often characterized by hyperconnectivity and heightened self-referential rumination among individuals diagnosed with depression. Nevertheless, the neurobiological responses elicited by CBT exhibit considerable variability across individuals, thereby highlighting the necessity for adjunctive methodologies that can augment or stabilize the neural remodeling induced by CBT.

### 3.3 Molecular and neuroplastic changes induced by CBT

Beyond the functional alterations within the brain, an expanding body of evidence suggests that CBT exerts influence over molecular and neuroplastic mechanisms. Numerous studies have documented an elevation in the brain-derived neurotrophic factor (BDNF) subsequent to CBT, which coincides with ameliorations in depressive symptomatology. In light of the pivotal function of BDNF in facilitating synaptic plasticity, neurogenesis, and neuronal viability (21, 22), these observations imply that psychotherapeutic interventions may activate fundamental biological pathways pertinent to antidepressant efficacy. Furthermore, CBT has been correlated with the normalization of HPA axis functionality, characterized by diminished cortisol secretion and enhanced responsiveness to stress. Given that chronic hyperactivation of the HPA axis is implicated in neurotoxicity, disrupted plasticity, and the persistence of depressive symptoms, the stress regulatory effects induced by CBT may constitute a critical biological mechanism underlying its therapeutic action (5, 23). Emerging empirical evidence further indicates that CBT may influence immune and inflammatory biomarkers, including diminished levels of pro-inflammatory cytokines such as interleukin-6 (IL-6) and tumor necrosis factor-α; however, the findings remain diverse and are affected by the clinical context (24). At the synaptic level, the behavioral activation and cognitive restructuring facilitated by CBT are likely to foster experience-dependent plasticity within neural networks that regulate mood. Nonetheless, the molecular and neuroplastic alterations induced by CBT are typically modest and may be inadequate to completely mitigate chronic neuroinflammation, oxidative stress, and monoaminergic dysregulation in cases of severe or protracted depression (25). This limitation underscores the justification for supplementary biological interventions such as *Lavandula angustifolia* and *Echium amoenum*, which can work synergistically to enhance neurotrophic, monoaminergic, and anti-inflammatory pathways in conjunction with CBT.

## 4 Rationale for integrating herbal medicine with psychotherapy

### 4.1 Concept of adjunctive and integrative treatments in psychiatry

Adjunctive and integrative therapeutic modalities have garnered heightened acknowledgment within the realm of contemporary psychiatry, signifying the escalating comprehension that MDD is a condition characterized by both biological and psychological heterogeneity (26). Traditional treatment frameworks that depend solely upon either pharmacological interventions or psychotherapeutic approaches frequently inadequately address the multidimensional characteristics of depression (27), especially in individuals exhibiting partial responses, residual symptomatology, or resistance to treatment. Consequently, integrative models that amalgamate psychological strategies with biological interventions are increasingly perceived as indispensable components rather than merely optional facets of individualized mental health care (28). In this framework, adjunctive therapy denotes the incorporation of supplementary interventions that augment the effectiveness of a principal treatment without supplanting it. CBT, despite its notable efficacy, predominantly influences cognitive and emotional processing networks through a top-down regulatory mechanism (29). Nevertheless, CBT in isolation may not adequately restore dysregulated neurobiological systems such as neuroinflammation, oxidative stress, monoaminergic imbalances, and compromised neurotrophic signaling (30). The integration of biologically active agents alongside psychotherapy represents a methodical strategy for simultaneously addressing both the psychological and molecular foundations of depression. Significantly, integrative methodologies correspond with the evolving paradigms of precision psychiatry, which underscore the importance of customizing interventions according to symptomatology, biological indicators, and individual patient preferences. When employed adjunctively, herbal medicines may serve as modulators of neurobiological susceptibility, whilst psychotherapy concurrently restructures maladaptive cognitive and behavioral patterns, thereby facilitating more enduring and holistic therapeutic outcomes.

### 4.2 Advantages of phytotherapy as a complementary approach

Phytotherapy presents numerous advantages as an adjunctive approach in the management of MDD (31). Herbal remedies encompass intricate assemblages of bioactive constituents that possess the capacity to engage multiple molecular targets concurrently, a characteristic that is particularly pertinent in light of the complex and multifactorial etiology of depressive disorders (32). In contrast to conventional synthetic pharmacological agents that operate on a singular target, phytochemicals can manifest pleiotropic effects, which encompass the modulation of monoaminergic neurotransmission, the augmentation of neurotrophic factors such as BDNF, the mitigation of neuroinflammation, and the alleviation of oxidative stress (32, 33). This multifaceted biological profile synergizes with the mechanisms underlying CBT, which primarily exerts influence over cognitive control networks and circuits involved in emotional regulation. By fostering neuroplasticity and diminishing the inflammatory and stress-related impediments to learning and adaptability, phytotherapeutic compounds may augment patients’ receptivity to psychotherapeutic modalities (32). From a clinical standpoint, herbal medicines may also represent a more palatable alternative for individuals hesitant to commence or persist with conventional antidepressant therapies due to apprehensions regarding adverse effects, societal stigma, or the risk of long-term dependency (34). Furthermore, numerous botanical interventions have exhibited anxiolytic and mood-regulating characteristics that are especially pertinent for coexisting anxiety manifestations, which often accompany depressive disorders and can hinder engagement in cognitive-behavioral therapy. When judiciously chosen and properly standardized, phytotherapeutic approaches may therefore provide a biologically supportive framework that enhances the cognitive and behavioral transformations advocated by psychotherapeutic practices.

### 4.3 Safety, tolerability, and patient adherence considerations

The considerations of safety and tolerability are paramount in the amalgamation of herbal medicine with psychotherapeutic practices. In comparison to traditional antidepressant medications, numerous herbal formulations when administered within scientifically substantiated dosage parameters, exhibit a diminished frequency of severe adverse effects and a lowered probability of sexual dysfunction, weight gain, or emotional desensitization. This advantageous tolerability profile may foster enhanced patient acceptance and sustained adherence over time, particularly among individuals who have previously encountered negative experiences with pharmacological interventions (35, 36). Augmented adherence is especially pertinent in psychotherapy-oriented interventions, where sustained engagement is essential for achieving therapeutic efficacy (37). Patients who regard their treatment as more “natural” or holistic may exhibit heightened motivation and commitment, thereby indirectly improving the outcomes of psychotherapy (38). However, it is imperative not to underestimate safety considerations. Potential interactions between herbs and pharmaceuticals, variability in product quality, and the absence of standardization continue to pose significant challenges that necessitate meticulous clinical oversight and thorough scientific investigation (39). From a clinical and translational perspective, the amalgamation of phytotherapy and psychotherapy ought to be informed by a systematic and evidence-based selection of phytotherapeutic agents, a comprehensive comprehension of their pharmacodynamic and pharmacokinetic characteristics, and unambiguous communication with patients.

## 5 *Lavandula angustifolia*: phytochemistry and neuropsychological effects

### 5.1 Bioactive compounds and pharmacokinetics

*Lavandula angustifolia* is among the most thoroughly researched phytotherapeutic species concerning neuropsychological interventions, attributed to its extensively delineated phytochemical composition and beneficial effects on the central nervous system (CNS). The neuropsychological repercussions are predominantly linked to volatile monoterpenes, which are complemented by non-volatile phenolic substances that collectively manifest anxiolytic, antidepressant-like, and neuroprotective properties. The principal bioactive components of *L. angustifolia* essential oil are linalool and linalyl acetate, which jointly contribute to the preponderance of its pharmacological efficacy (40). Linalool has been demonstrated to influence GABAergic neurotransmission, thereby augmenting inhibitory signaling and diminishing neuronal excitability without provoking the significant sedation commonly associated with traditional anxiolytics (41, 42). These characteristics hold particular significance in the context of depressive disorders, wherein increased stress reactivity and emotional dysregulation often coexist with cognitive disturbances. Beyond the GABAergic mechanisms, the constituents of lavender exert an influence on monoaminergic pathways that are implicated in the regulation of mood. Preclinical investigations reveal that linalool possesses the capacity to modulate serotonergic and dopaminergic transmission, thereby contributing to antidepressant-like outcomes in behavioral models (43). Notably, these effects seem to involve functional modulation as opposed to direct receptor agonism, which implies a diminished risk of receptor desensitization with prolonged administration. Such characteristics affirm the appropriateness of *L. angustifolia* as an adjunctive agent rather than a substitute for established antidepressant or psychotherapeutic treatments. Pharmacokinetic studies suggest that the volatile constituents of lavender are swiftly absorbed subsequent to either oral or inhalational administration. Owing to their lipophilic properties, linalool and analogous terpenes can readily traverse the blood-brain barrier, achieving measurable CNS concentrations within a brief time period (44). This expeditious bioavailability may account for the relatively rapid onset of anxiolytic and calming effects observed in clinical contexts, potentially enhancing early engagement and emotional regulation during CBT. In conjunction with volatile terpenoids, *Lavandula angustifolia* is composed of phenolic acids and flavonoids, such as rosmarinic acid and apigenin derivatives, which have been demonstrated to possess antioxidant and anti-inflammatory properties (45). Despite being present in lower concentrations compared to essential oil constituents, these compounds may play a significant role in providing long-term neuroprotective benefits by mitigating oxidative stress and neuroinflammation—pathophysiological processes increasingly acknowledged as contributors to the etiology of MDD. In summary, the phytochemical profile and pharmacokinetic properties of *Lavandula angustifolia* substantiate its function as a neuromodulatory and stress-mitigating agent rather than merely a sedative. Its ability to modulate inhibitory neurotransmission, influence monoaminergic signaling, and restore inflammatory equilibrium offers a mechanistic rationale for its incorporation into psychotherapeutic frameworks. By fostering a neurobiological milieu that is favorable for emotional regulation and synaptic plasticity, *Lavandula angustifolia* may augment the effectiveness of cognitive-behavioral therapy and other psychotherapeutic interventions in individuals diagnosed with MDD.

### 5.2 Antidepressant and anxiolytic effects: clinical evidence

**Figure 2:**
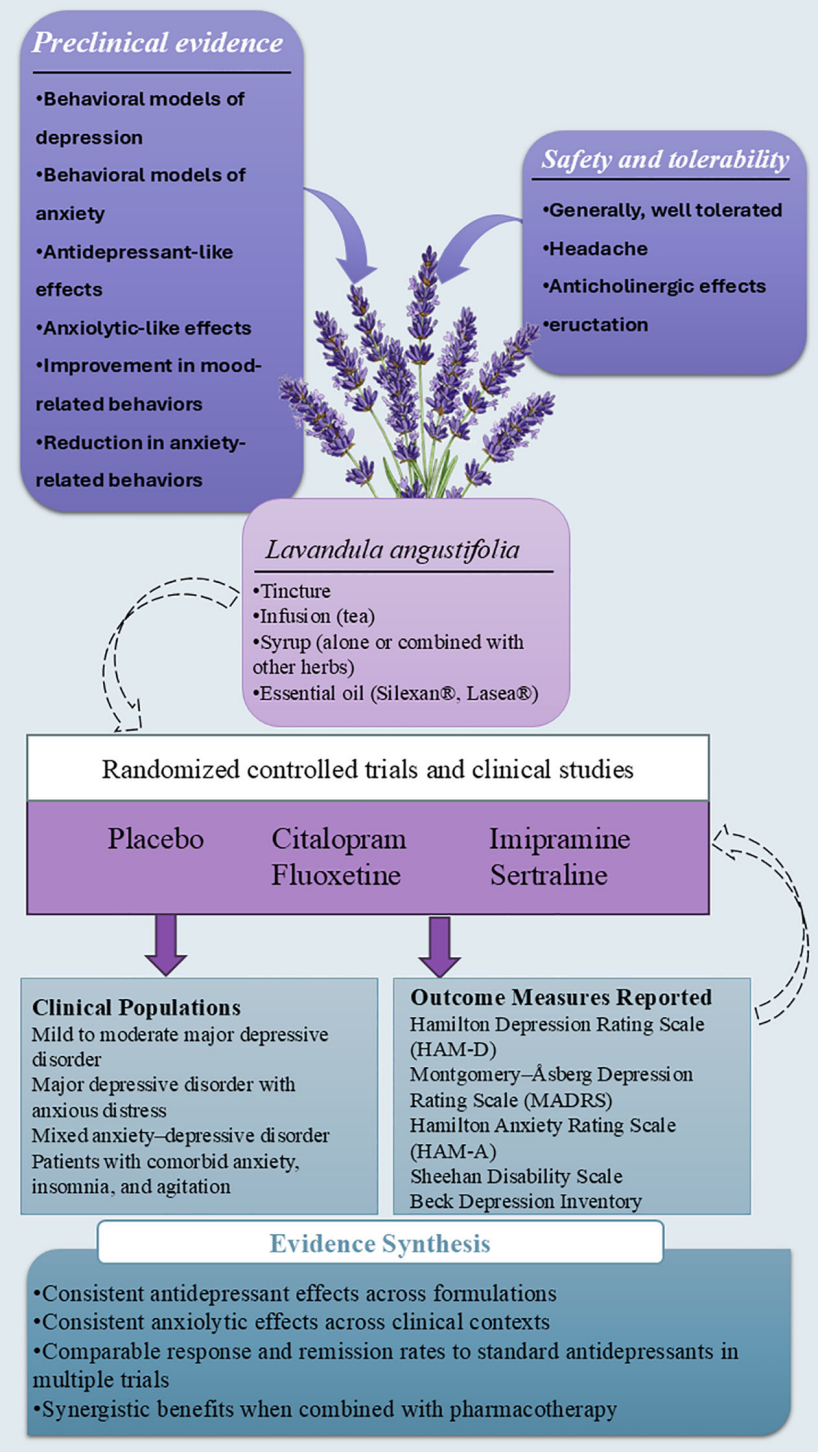
Summary of clinical evidence supporting the antidepressant and anxiolytic effects of *Lavandula angustifolia*. Across animal models and randomized clinical trials, multiple formulations of *L. angustifolia* demonstrate consistent reductions in depressive and anxiety symptoms. Clinical studies report efficacy comparable to conventional antidepressants in mild to moderate depression, favorable tolerability, and enhanced outcomes when used adjunctively, supporting its potential role as a complementary intervention in mood disorders.

The comprehension of the antidepressant and anxiolytic properties of *Lavandula angustifolia* holds considerable clinical and scientific significance, particularly in light of the widespread occurrence of depressive and anxiety disorders coupled with the limitations inherent in existing therapeutic modalities. Numerous patients encounter insufficient therapeutic responses or adverse effects stemming from traditional pharmacological interventions, thereby highlighting the necessity for safer and more tolerable alternatives or adjunctive therapies. A double-blind, randomized clinical investigation evaluated the therapeutic efficacy of a combined lavender–dodder herbal syrup in conjunction with citalopram among individuals diagnosed with MDD characterized by anxious distress. During a six-week intervention period, participants were administered either citalopram (20 mg/day) alongside a placebo syrup or lavender–dodder syrup (5 mL administered bi-daily) in conjunction with placebo tablets. The severity of depression and anxiety was measured utilizing the Hamilton Depression and Anxiety Rating Scales at baseline, as well as at weeks three and six. Both treatment cohorts exhibited significant reductions in depression scores over the duration of the study, with no statistically significant differences detected between the interventions. Anxiety scores did not demonstrate any significant disparities between groups at week three; however, at week six, a markedly greater reduction was documented in the herbal syrup cohort. Secondary outcomes, which encompassed treatment response and remission rates, were found to be comparable across both groups (46) (**Table 1**; **Figure 2****).**

**Table 1:** Clinical evidence of *Lavandula angustifolia* and related herbal interventions in major depressive disorder.

Study	Sample size	Intervention (dose + extract type)	Comparator	Outcome scale	Results	P-value	Limitations	Ref
Lavender–dodder syrup RCT	n=56	Lavender–dodder herbal syrup (5 mL every 12 h) vs citalopram 20 mg/day (6-week RCT)	Citalopram 20 mg/day	HAM-D, HAM-A	Depression: ↔ (no significant between-group difference)/Anxiety: ↓ (greater reduction in herbal group at week 6)	HAM-D: P = 0.61 (between-group difference)/HAM-A: P = 0.007 (week 6 between-group difference)	Small sample size; short duration; lack of long-term follow-up	(46)
Lavandula tincture vs imipramine	n=45	*Lavandula angustifolia* tincture 60 drops/day (1:5 in 50% ethanol) ± imipramine 100 mg/day (4-week RCT)	Imipramine 100 mg/day	HAM-D	Lavender alone: ↓ (less effective than imipramine)/Combination: ↓↓ (greater improvement than imipramine alone)	HAM-D change: P = 0.001 (lavender vs imipramine)/HAM-D change: P<0.0001 (combination vs imipramine)	Small sample size; short duration; single-center design	(47)
Melissa officinalis + Lavandula vs fluoxetine	n=45	*Lavandula angustifolia* 2 g/day or *Melissa officinalis* 2 g/day vs fluoxetine 20 mg/day (8-week RCT)	Fluoxetine 20 mg/day	HAM-D (17-item)	Herbal vs fluoxetine: ↔ (comparable antidepressant effect)	HAM-D score change: P = 0.877 (between-group difference)	No placebo group; small sample size; limited standardization of extracts	(48)
*Lavandula angustifolia* infusion + citalopram vs citalopram alone	n=80	*Lavandula angustifolia* infusion (5 g dried plant, 2 cups/day) + citalopram 20 mg vs citalopram 20 mg (8-week RCT)	Citalopram 20 mg/day	HAM-D	Depression: ↓ (greater reduction in combination group vs control at both time points)	Week 4: P<0.05 (HAM-D score difference)/Week 8: P<0.01 (HAM-D score difference)	Single-center study; limited blinding details; short follow-up	(49)
Silexan vs sertraline vs placebo in MDD	n=498	Silexan 80 mg/day vs sertraline 50 mg/day vs placebo (56-day RCT)	Placebo; Sertraline	MADRS, SDS	Depression: ↓ (Silexan and sertraline superior to placebo); Functional impairment: ↓	MADRS: P<0.01 (Silexan vs placebo)/SDS: P<0.001 (functional improvement vs placebo)	Industry-supported trial; limited long-term outcomes	(50)
Silexan in major depressive episode (Phase III trial)	n=498	Silexan 80 mg/day vs sertraline 50 mg/day vs placebo (8-week RCT)	Placebo; Sertraline	MADRS, PHQ-9, BDI, CGI, SDS	Depression: ↓ (Silexan > placebo; similar to sertraline); Response/remission: ↑	MADRS: P<0.01 (Silexan vs placebo)/Response & remission: P<0.05 (vs placebo)	Short duration; heterogeneity in depressive severity; limited mechanistic biomarkers	(51)
Lasea^®^ case series	n=8	Lavender oil capsules (Lasea^®^, dose not specified clinical routine)	None (observational)	HAM-D	↓ Depression; ↓ anxiety; ↓ insomnia symptoms	P<0.05	Very small sample; case series design; no control group	(52)
Silexan in MADD	n=318	Silexan 80 mg/day	Placebo	HAM-A, MADRS	↓ Anxiety; ↓ depression; ↑ quality of life	HAMA: P<0.01; MADRS: P<0.001 (between-group differences)	Short duration (70 days); mixed disorder population	(53)
Silexan functional impairment	n=498	Silexan 80 mg/day	Placebo/sertraline	SDS scale	↑ Functional improvement; ↓ disability	P<0.001 (SDS total score vs placebo)	*Post-hoc* functional analysis; short follow-up	(54)
Lavandula tincture ± imipramine RCT	n=45	*Lavandula angustifolia* tincture 60 drops/day (1:5 hydroalcoholic extract) alone or + imipramine	Imipramine 100 mg/day	HAM-D	↓ Less effective than imipramine alone; ↑ when combined with imipramine	P=0.001 → tincture vs imipramine; P<0.0001 → combination vs imipramine	Small sample; short (4 weeks); no long-term follow-up	(55)
Lavandula + Dracocephalum adjunct to sertraline	n=70	*Lavandula angustifolia* + *Dracocephalum ruyschiana* syrup (traditional unit dose, 3×/day) + sertraline 100 mg/day	Sertraline 100 mg/day	BDI	↓ Greater reduction in depression in adjunct group	P=0.02 → between-group difference after 6 weeks	No placebo group; non-standardized extract composition; moderate follow-up (6 weeks)	(56)

RCT, randomized controlled trial; HAM-D, Hamilton Depression Rating Scale; HAM-A, Hamilton Anxiety Rating Scale; MADRS, Montgomery–Åsberg Depression Rating Scale; BDI, Beck Depression Inventory; SCID-IV, Structured Clinical Interview for DSM-IV; DSM-5, Diagnostic and Statistical Manual of Mental Disorders, Fifth Edition; MDD, major depressive disorder; MADD, mixed anxiety–depressive disorder; SDS, Sheehan Disability Scale; PHQ-9, Patient Health Questionnaire-9; L., *Lavandula angustifolia*; E., *Echium amoenum*; H., Hypericum perforatum; D., Dracocephalum ruyschiana.

Another study, the therapeutic efficacy of *Lavandula angustifolia* tincture was systematically compared with that of imipramine and assessed as an adjunctive treatment in adult patients diagnosed with mild to moderate MDD. A total of forty-five outpatients who fulfilled the DSM-IV diagnostic criteria for major depression, exhibiting baseline Hamilton Rating Scale for Depression (HAMD) scores of 18 or higher, were randomly allocated to receive either lavandula tincture (60 drops per day) in conjunction with a placebo tablet, imipramine (100 mg per day) accompanied by a placebo drop, or a combination of imipramine (100 mg per day) and lavandula tincture over a four-week duration. The severity of depressive symptoms was meticulously evaluated throughout the course of the study. Lavandula tincture administered as a monotherapy demonstrated a lower efficacy compared to imipramine (F = 13.16, df=1, P = 0.001). The occurrence of adverse effects varied among the treatment groups, with anticholinergic side effects being significantly more prevalent in the imipramine cohort, while headaches were reported with greater frequency in the lavandula group. The synergistic application of imipramine and lavandula tincture yielded a more pronounced and expedited reduction in depressive symptoms relative to imipramine monotherapy (F = 20.83, df=1, P<0.0001) (47). An investigation, the therapeutic efficacy of Melissa officinalis (2 g/day) and *Lavandula angustifolia* (2 g/day) was systematically evaluated against fluoxetine (20 mg/day) in a cohort of adults diagnosed with mild to moderate MDD. A total of forty-five outpatients who fulfilled the DSM-5 diagnostic criteria for major depression were randomly allocated to one of the three intervention groups. The severity of depressive symptoms was systematically assessed at baseline and at weeks 2, 4, and 8 utilizing the 17-item HAM-D. Both herbal interventions resulted in significant reductions in depressive symptomatology that were comparable to those observed with fluoxetine, with no statistically significant differences noted between groups (F = 0.131, df=2,42, P = 0.877). These results imply that M. officinalis and *L. angustifolia* may possess potential antidepressant properties in cases of mild to moderate depression. Identified limitations encompassed the lack of a placebo comparison group and the relatively small sample size, underscoring the necessity for more extensive, controlled studies to validate these findings and elucidate their clinical applicability (48).

A randomized clinical investigation assessed the impact of *Lavandula angustifolia* infusion on depressive manifestations in individuals undergoing treatment with citalopram. Eighty outpatients, formally diagnosed with MDD through structured interviews and evaluated using the HAMD, were randomly allocated to either a conventional treatment cohort receiving citalopram at a dosage of 20 mg/day or an experimental cohort receiving citalopram at the same dosage in conjunction with two cups per day of *L. angustifolia* infusion, derived from 5 g of dried flowers. The levels of depressive symptoms and any treatment-related adverse effects were evaluated at the four and eight-week marks. Following the four-week period, the average depression score recorded was 17.5 ± 3.5 for the standard treatment group and 15.2 ± 3.6 for the experimental group (P<0.05). At the eight-week assessment, the scores were noted to be 16.8 ± 4.6 and 14.8 ± 4, respectively (P<0.01). No statistically significant differences in adverse effects were identified between the two groups (49). The aim of study was to investigate the antidepressant efficacy of silexan, an anxiolytic compound derived from *Lavandula angustifolia*, in individuals diagnosed with mild to moderate MDD. A cohort consisting of 498 participants, characterized by MADRS scores ranging from 19 to 34, was systematically randomized to receive either silexan at a dosage of 80 mg per day, sertraline at a dosage of 50 mg per day, or a placebo over a duration of 56 days. The primary endpoint of this investigation was the alteration in MADRS total score from baseline to the conclusion of the treatment period. Upon completion of the eight-week intervention, both silexan and sertraline exhibited significant reductions in MADRS scores when contrasted with the placebo group, yielding absolute differences of 2.17 (95% CI: 0.58–3.76) and 2.59 (95% CI: 1.02–4.17) points, respectively (P<0.01). Moreover, silexan was found to significantly ameliorate functional impairment as assessed by the Sheehan Disability Scale (difference 2.40, 95% CI: 1.04–3.76; P<0.001). Both pharmacological interventions were well tolerated among the participants, with eructation being identified as the most prevalent adverse effect associated with silexan (50).

A phase III, randomized clinical trial investigated the antidepressant efficacy of Silexan, an essential oil derived from *Lavandula angustifolia*, in adults experiencing mild to moderate major depressive episodes, as indicated by a MADRS score ranging from 19 to 34. A total of 498 participants were randomly assigned to receive either Silexan at a dosage of 80 mg/day, sertraline at a dosage of 50 mg/day, or a placebo, administered once daily over a duration of eight weeks. The primary outcome measure was the change in the total MADRS score from baseline to week 8, while secondary outcome measures included response rates (defined as a ≥50% reduction in MADRS), remission (MADRS score <10), as well as scores on the Patient Health Questionnaire-9 (PHQ-9), Beck Depression Inventory (BDI), Clinical Global Impressions, and Sheehan Disability Scale. The adjusted mean reductions in MADRS scores were found to be 12.1 points for Silexan, 12.6 points for sertraline, and 9.95 points for placebo, with both active treatment groups exhibiting statistically significant superiority over the placebo condition (P<0.01). The response rates were determined to be 53.5% for Silexan and 54.0% for sertraline in comparison to 41.5% for the placebo group; remission rates were recorded at 44.4%, 45.2%, and 32.6%, respectively (51). The retrospective case series evaluated the therapeutic efficacy of Lasea^®^, a lavender oil encapsulation, in individuals diagnosed with MDD who concurrently exhibit symptoms of anxiety, insomnia, and psychomotor agitation. Eight clinical cases managed at the Department of Psychiatry, Charité-Universitätsmedizin Berlin, were scrutinized for factors such as dosage, duration of treatment, adverse effects, and clinical outcomes. The efficacy was appraised by analyzing alterations in the HAMD-17 total score and its respective subscores. In six instances, the adjunctive use of Lasea^®^ with an antidepressant resulted in a significant reduction of depressive symptomatology. Lasea^®^ demonstrated a reduction in psychomotor agitation in six cases, alleviated psychological anxiety in five cases, mitigated somatic anxiety in four cases, and enhanced both sleep-onset and sleep-maintenance insomnia in three cases each. The results indicate that Lasea^®^ may serve as a beneficial adjunct in alleviating anxiety-related symptoms, agitation, and sleep disturbances in patients with MDD, thereby contributing to an overall clinical improvement when integrated with conventional antidepressant treatment (52).

A research assessed the therapeutic efficacy of Silexan, a phytotherapeutic essential oil derived from *Lavandula angustifolia*, in a cohort of adults diagnosed with mixed anxiety and depressive disorder (MADD; ICD-10 F41.2). A total of 318 outpatients exhibiting Hamilton Anxiety Rating Scale (HAMA) scores of ≥18, indicative of a moderately severe anxious and depressive affect, were randomly assigned to receive either Silexan at a dosage of 80 mg per day or a placebo over a duration of 70 days. The primary endpoints of this study were the alterations in total scores on the HAMA and Montgomery and Asberg Depression Rating Scale (MADRS). The HAMA scores exhibited a reduction of 10.8 ± 9.6 points in the Silexan group compared to an 8.4 ± 8.9 point reduction in the placebo group (P<0.01), while the MADRS scores demonstrated a decline of 9.2 ± 9.9 points versus 6.1 ± 7.6 points, respectively (P<0.001). Furthermore, Silexan was found to enhance daily functioning and health-related quality of life metrics. Notably, eructation was identified as the sole adverse event with a higher incidence in the Silexan treatment group (53). Another study evaluated the efficacy of Silexan, a phytotherapeutic agent derived from *Lavandula angustifolia*, in ameliorating functional impairments in adults experiencing mild to moderate major depressive episodes, as quantified by the Montgomery-Asberg Depression Rating Scale (MADRS) with scores ranging from 19 to 34. A total of 498 participants were randomly allocated to receive either Silexan at a dosage of 80 mg per day, sertraline at a dosage of 50 mg per day, or a placebo over a period of eight weeks. Functional outcomes were assessed utilizing the Sheehan Disability Scale (SDS), which encompassed total scores as well as specific domains related to work, social life/leisure, and family/home responsibilities, in addition to measures of lost (DL) and underproductive days (DU). The administration of Silexan resulted in a statistically significant enhancement of SDS total scores in comparison to placebo (adjusted mean difference of 2.40, P<0.001), with notable improvements observed in each SDS subdomain (P<0.05). Furthermore, DL and DU exhibited a reduction in comparison to the placebo group (P = 0.055 and 0.011, respectively). These enhancements in functional capacity were paralleled by significant reductions in MADRS scores (P<0.01), suggesting that Silexan not only alleviates depressive symptoms but also fosters improvements in daily functional capabilities (54).

In a four-week, double-blind, single-center randomized clinical trial, the therapeutic efficacy of *Lavandula angustifolia* tincture was systematically compared with that of imipramine and assessed as an adjunctive treatment in adults diagnosed with mild to moderate major depression (baseline HAM-D ≥18). A total of forty-five outpatients who fulfilled the DSM-IV criteria were randomly allocated to receive either lavandula tincture (1:5 in 50% alcohol, 60 drops/day) in conjunction with a placebo tablet, imipramine at a dosage of 100 mg/day paired with a placebo drop, or imipramine at 100 mg/day combined with lavandula tincture. The administration of lavandula tincture in isolation proved to be significantly less efficacious compared to imipramine (F = 13.16, df=1, P = 0.001). Notable variations in adverse effects were observed between the treatment groups: anticholinergic side effects were more prevalent in the imipramine group, whereas headaches were reported more frequently among participants receiving lavandula. The synergistic effect of combining imipramine with lavandula tincture resulted in more pronounced and earlier alleviations of depressive symptoms compared to the administration of imipramine alone (F = 20.83, df=1, P<0.0001). These results indicate that lavandula tincture may possess significant therapeutic potential as an adjunctive treatment for individuals experiencing mild to moderate depression (55). The aim of another study was to determine the impact of *Lavandula angustifolia* and *Dracocephalum ruyschiana* extracts as adjunctive treatments to sertraline in adults diagnosed with mild to moderate MDD. Seventy subjects were randomly allocated to receive either sertraline at a dosage of 100 mg/day accompanied by a placebo syrup or sertraline at a dosage of 100 mg/day in conjunction with a herbal syrup comprising two units of *L. angustifolia* extract and one unit of D. ruyschiana extract administered three times daily. The severity of depression was evaluated through the Structured Clinical Interview for DSM-IV (SCID-IV) and the Beck Depression Inventory (BDI) at both baseline and a six-week interval. Baseline BDI scores were comparable across the groups (P>0.05). Following the six-week period, the experimental cohort exhibited statistically significant enhancements in BDI scores relative to the control cohort (P = 0.02), suggesting that the integration of herbal extracts may potentiate the antidepressant efficacy of sertraline (56). Overall, these investigations elucidated that *Lavandula angustifolia*, either in isolation or in conjunction with botanicals such as *Echium amoenum*, *Melissa officinalis*, and *Dracocephalum ruyschiana*, or alongside conventional antidepressant pharmacotherapy, manifests both antidepressant and anxiolytic properties in individuals experiencing mild to moderate MDD and mixed anxiety-depressive states. From a mechanistic perspective, these effects are attributed to the modulation of monoaminergic neurotransmission, particularly within serotonergic and dopaminergic pathways, thereby enhancing both mood regulation and cognitive-emotional processing. Additionally, empirical evidence substantiates neurotrophic effects, likely through the upregulation BDNF and associated neuroplasticity pathways, alongside anti-inflammatory and antioxidant mechanisms that mitigate cytokine-mediated neuroinflammation and oxidative stress. Functional enhancements are positively correlated with decreases in scores from the MADRS, HAM-D, and HAM-A. The adjunctive employment of standard antidepressants or CBT reveals synergistic outcomes, expediting symptom alleviation and augmenting response and remission rates. Collectively, these findings underscore a multi-targeted molecular profile, endorsing *L. angustifolia* and related herbal entities as promising integrative modalities for the personalized management of MDD.

### 5.3 Critical appraisal of clinical evidence and methodological limitations

Notwithstanding the encouraging therapeutic indicators documented in various studies, numerous significant constraints must be acknowledged when evaluating the extant evidence. A considerable number of clinical trials are characterized by relatively limited sample sizes, frequently ranging from approximately 40 to 80 participants, which may constrain statistical power and elevate the likelihood of type II error. Moreover, the majority of studies utilize brief intervention durations, generally spanning from 4 to 8 weeks, consequently limiting inferences regarding long-term efficacy and the prevention of relapse. Additionally, there exists substantial heterogeneity in study designs and intervention protocols. Various formulations of *Lavandula angustifolia* have been utilized, encompassing tinctures, infusions, and standardized preparations such as Silexan, alongside combination herbal products (e.g., lavender in conjunction with dodder or other medicinal botanicals). This variability renders direct comparison across studies problematic and restricts the generalizability of the findings. Methodological constraints are also apparent in numerous trials, characterized by single-center designs, the absence of placebo-controlled groups in certain studies, and reliance on subjective outcome assessments such as Hamilton Depression Rating Scale scores that lack consistent biomarker validation. Although some large-scale, rigorously designed RCTs employing standardized formulations (e.g., Silexan) yield more substantial evidence, many smaller investigations should be regarded as preliminary in nature. Furthermore, the majority of the extant clinical evidence assesses these herbal interventions either as standalone therapies or as adjuncts to pharmacological treatments (e.g., imipramine, sertraline, or citalopram), rather than in conjunction with psychotherapeutic methodologies such as CBT. This illustrates a significant void in the existing literature and emphasizes that the proposed integration with CBT remains predominantly theoretical. Collectively, these limitations accentuate the necessity for meticulously designed, large-scale, multicenter RCTs utilizing standardized formulations, extended follow-up periods, and integrative methodologies that explicitly evaluate combination strategies with psychotherapeutic interventions.

## 6 *Echium amoenum*: phytochemistry and neuropsychological effects

### 6.1 Bioactive constituents and pharmacological properties

*Echium amoenum* is a medicinal flora that has been conventionally employed in Iranian and Middle Eastern medical practices for the alleviation of mood disturbances, anxiety, and sleep-related disorders. In recent decades, there has been a burgeoning of experimental and clinical interest pertaining to its neuropsychological effects, which are ascribed to a unique phytochemical composition characterized by polyunsaturated fatty acids, phenolic compounds, and bioactive flavonoids. One of the most notable bioactive constituents of *E. amoenum* is γ-linolenic acid (GLA), an omega-6 polyunsaturated fatty acid extracted from its seed oil. GLA functions as a precursor for anti-inflammatory eicosanoids and has been demonstrated to influence membrane fluidity, neurotransmitter release, and neuroinflammatory signaling (57). Beyond its fatty acid composition, the blossoms of *E. amoenum* are enriched with phenolic acids and flavonoids, such as rosmarinic acid, cyanidin derivatives, and delphinidin glycosides, which play integral roles in its antioxidant and neuroprotective properties (58, 59). These bioactive compounds proficiently neutralize reactive oxygen species and impede lipid peroxidation, consequently safeguarding neuronal membranes against oxidative injury. Experimental investigations further indicate that *E. amoenum* demonstrates anti-inflammatory properties via the downregulation of pro-inflammatory cytokines and the modulation of cyclooxygenase and lipoxygenase pathways (11). From a pharmacological perspective, *E. amoenum* extracts have exhibited anxiolytic and antidepressant-like effects in preclinical animal models, correlated with enhancements in behavioral despair and stress-induced responses (60). Moreover, limited clinical trials lend additional support to its mood-enhancing and anxiolytic capabilities in individuals experiencing mild to moderate depression, with positive tolerability profiles documented during short-term applications. Significantly, these effects are not correlated with sedation or cognitive deficits, indicating that *E. amoenum* may facilitate emotional equilibrium without disrupting cognitive functions that are critical for psychotherapeutic interactions. Collectively, the bioactive components of *E. amoenum* provide a multifaceted pharmacological profile that includes anti-inflammatory, antioxidant, and neuromodulatory characteristics. This profile is consistent with modern conceptualizations of depression that underscore neuroimmune dysfunction and compromised neuroplasticity.

### 6.2 Evidence from clinical studies in depression

*Echium amoenum*, a traditional medicinal flora indigenous to Iran, has exhibited significant antidepressant and anxiolytic effects in both preclinical and clinical investigations. In experimental animal models, extracts of *E. amoenum* led to a decrease in immobility during the forced swim and tail suspension assessments, thereby suggesting antidepressant-like properties, which may be facilitated through the modulation of the monoaminergic system, reduction of oxidative stress, and anti-inflammatory mechanisms. Mechanistic explorations indicate an upregulation of BDNF alongside the normalization of hyperactivity within the HPA axis. In clinical contexts, RCTs have demonstrated that supplementation with *E. amoenum* markedly enhances depressive symptoms and anxiety metrics, whether utilized as a standalone intervention or as an adjunct to conventional antidepressant therapies, all while maintaining a commendable safety profile. In a parallel randomized controlled trial, a cohort of fifty individuals diagnosed with MDD in accordance with the DSM-5 criteria were systematically allocated to receive either *Echium amoenum* compound syrup (EACS) or citalopram for a duration of eight weeks. The efficacy of the treatments and the incidence of recurrence were assessed utilizing the Hamilton Depression Rating Scale at baseline, as well as at weeks four, eight, and twelve. Both study groups exhibited statistically significant reductions in depression scores across the evaluated time periods. At the conclusion of week eight, the mean Hamilton scores for the EACS cohort were found to be significantly lower in comparison to those recorded for the citalopram cohort (P = 0.03). In terms of adverse effects, it was observed that 52% of participants in the citalopram group reported experiencing complications, whereas only 12% of individuals in the EACS group encountered such issues, thereby suggesting a substantially improved tolerability profile for the herbal intervention (61) (**Table 2****).** In a six-week, double-blind, parallel-group clinical trial, thirty-five individuals diagnosed with mild to moderate MDD (HAM-D score ≥18) were systematically allocated to either receive 375 mg of aqueous extract of *Echium amoenum* daily or a placebo. Participants were assessed at baseline (week 0), as well as at weeks 1, 2, 4, and 6 utilizing the Hamilton Depression Rating Scale (HAM-D17), the HAM-A14, and a checklist for adverse effects. By the fourth week, subjects administered the *E. amoenum* extract exhibited a statistically significant decrease in depressive symptoms when juxtaposed with those receiving the placebo. No statistically significant disparities in anxiety scores were detected between the two groups. Adverse effects reported encompassed headache, somnolence, vomiting, dry mouth, constipation, and blurred vision; nonetheless, the frequency of these adverse effects did not show significant variation between the extract and placebo cohorts (62).

**Table 2:** Clinical trials investigating the efficacy and safety of *Echium amoenum* in patients with depression.

Study	Sample size	Intervention (dose + extract type)	Comparator	Outcome scale	Results	P-value	Limitations	Ref
*Echium amoenum* compound syrup vs citalopram RCT	n=50	*Echium amoenum* compound syrup (EACS), oral; 8 weeks	Citalopram tablet	HAM-D	↓ Depression in both groups; ↑ greater reduction in EACS vs citalopram; ↓ adverse effects in EACS	P=0.03 → between-group difference at week 8 (HAM-D score)	Small sample; short follow-up; limited mechanistic/biomarker data	(61)
Aqueous *Echium amoenum* extract vs placebo	n=35	*Echium amoenum* aqueous extract 375 mg/day (oral), 6 weeks	Placebo	HAM-D17, HAM-A14	↓ Depression (significant at week 4); ↔ anxiety (no significant change)	P<0.05 → depressive symptom reduction vs placebo (week 4)	Small sample; short duration; limited safety evaluation detail	(62)
*Echium amoenum* + Hypericum perforatum vs fluoxetine	n=51	*E. amoenum* + *Hypericum perforatum* extract 350 mg twice daily (oral), 8 weeks	Fluoxetine 20 mg/day	HAM-D	↔ Similar antidepressant effect vs fluoxetine; ↓ side effects (dry mouth) in herbal group	P>0.05 → between-group efficacy comparison; P<0.05 → side effect difference	No placebo group; combination herbal intervention prevents attribution of effect to single herb	(63)
*Echium amoenum* vs fluoxetine in menopausal women RCT	n=72	*Echium amoenum* hydroalcoholic extract capsules (8 weeks)	Fluoxetine capsule	HAM-D	↔ No difference vs fluoxetine at end of study; ↓ depression over time in both groups; ↑ early improvement in herbal group	P=0.889 baseline (no difference); P ≤ 0.01 week 4 (between-group); P = 0.1 week 8 (no difference); P<0.001 (within-group improvement in *E. amoenum*)	No placebo group; population limited to menopausal women; short follow-up	(64)
Aqueous *Echium amoenum* vs placebo	n=35	*Echium amoenum* aqueous extract 375 mg/day (oral), 6 weeks	Placebo	HAM-D	↓ Depression vs placebo (significant at week 4); ↔ no significant difference at week 6; ↑ overall safety (no added adverse effects)	P=0.018 → between-group difference at week 4; non-significant at week 6	Small sample; short duration; marginal late significance	(65)

RCT, randomized controlled trial; HAM-D, Hamilton Depression Rating Scale; HAM-A, Hamilton Anxiety Rating Scale; MDD, major depressive disorder; DSM-5, Diagnostic and Statistical Manual of Mental Disorders, Fifth Edition; EACS, *Echium amoenum* compound syrup; *E. amoenum*, *Echium amoenum*; H. perforatum, Hypericum perforatum; E., Echium; HAM-D17, 17-item Hamilton Depression Rating Scale.

Another eight-week double-blind, parallel-group clinical trial, a total of 51 individuals presenting with mild to moderate depressive symptoms were randomly allocated to receive either a regimen of 20 mg fluoxetine or 350 mg of a composite herbal extract derived from *Echium amoenum* and *Hypericum perforatum* administered bi-daily. The severity of depression was quantitatively evaluated utilizing the HAMD at baseline (week 0), as well as at four and eight weeks’ post-initiation of the intervention. The findings revealed no statistically significant disparities in HAM-D scores between the herbal extract group and the fluoxetine cohort at both weeks four and eight (P>0.05), thereby suggesting equivalent antidepressant efficacy between the two treatment modalities. Xerostomia was identified as the sole adverse effect documented, occurring with notably lower frequency in the herbal treatment group at weeks two and four (P<0.05). Additional side effects were observed to be minimal and did not exhibit significant differences between the two groups (63). In addition, a cohort of 72 menopausal women diagnosed with depressive disorders was systematically allocated to receive either an extract of *Echium amoenum* or fluoxetine over a duration of 8 weeks. The severity of depressive symptoms was assessed utilizing the HAMD at the outset of the study and subsequently at weeks 4, 6, and 8. At the initial assessment, the mean depression scores demonstrated comparable values between the *Echium amoenum* cohort (19 ± 0.477) and the fluoxetine cohort (18.9 ± 0.440, P = 0.889). By the conclusion of the 4th week, the *Echium amoenum* cohort exhibited a statistically significant decrease in depression scores when compared to baseline measurements (P ≤ 0.01). At the termination of the study, no statistically significant disparity was noted between the two groups (P = 0.1). Collectively, the participants administered with the *Echium amoenum* extract displayed a marked reduction in depression scores throughout the intervention period (P<0.001), with no adverse effects reported (64).

In a randomized, double-blind clinical trial conducted over a duration of six weeks, thirty-five individuals diagnosed with mild to moderate MDD (HAMD≥18) were systematically allocated to receive either 375 mg of an aqueous extract of *Echium amoenum* or a placebo. The severity of depressive symptoms was assessed at baseline and at weeks 1, 2, 4, and 6 utilizing the Hamilton Depression Rating Scale. By week 4, the cohort receiving *Echium amoenum* exhibited a statistically significant decrease in depressive scores in comparison to the placebo group (P = 0.018). By the conclusion of week 6, although the observed difference persisted, it was rendered marginally non-significant. The administration of the extract did not result in a higher incidence of adverse effects than that observed in the placebo group throughout the duration of the study. Collectively, these findings indicated that the aqueous extract of *Echium amoenum* yields a substantial reduction in depressive symptoms throughout the treatment interval, thereby indicating both therapeutic efficacy and safety in individuals with mild to moderate MDD, underscoring the necessity for further investigations involving larger sample sizes and prolonged follow-up periods (65). Overall, *Echium amoenum* demonstrates antidepressant properties through various mechanisms. Its bioactive constituents, comprising flavonoids and phenolic compounds, influence serotonergic and dopaminergic neurotransmission, thereby enhancing mood and alleviating depressive symptoms. Furthermore, the herb exhibits anxiolytic characteristics, presumably through the modulation of the HPA axis and the mitigation of stress-induced corticosterone secretion. In addition, *Echium amoenum* is endowed with antioxidant and anti-inflammatory properties, which contribute to the reduction of oxidative stress and neuroinflammation linked to depression. Clinical investigations indicate notable decreases in scores on the Hamilton Depression Rating Scale, which are comparable to or slightly superior to those associated with conventional antidepressants, while eliciting fewer adverse effects. These results imply that *Echium amoenum* may represent a safe and effective alternative or supplementary option to traditional pharmacological interventions for individuals experiencing mild to moderate depression.

### 6.3 Critical appraisal of clinical evidence and methodological limitations

Despite promising results reported in various clinical trials, the evidence substantiating the antidepressant properties of *Echium amoenum* necessitates cautious interpretation owing to numerous methodological constraints. The majority of existing studies exhibit relatively limited sample sizes, typically fluctuating between 35 to 72 participants, thereby constraining statistical power and undermining the robustness of the derived conclusions. Furthermore, the duration of the interventions is predominantly brief (ranging from 4 to 8 weeks), which inhibits the assessment of long-term efficacy, prevention of recurrence, and the maintenance of clinical benefits over time. A significant degree of heterogeneity is also evident in study designs and herbal formulations, encompassing aqueous extracts, hydroalcoholic preparations, and multi-component herbal syrups (for instance, combinations with Hypericum perforatum or other traditional formulations). This variability diminishes the comparability among studies and complicates the interpretation of overall efficacy. Methodological issues are further manifested in numerous trials, including single-center methodologies, insufficient documentation of allocation concealment in earlier research, and dependence on symptom-based assessment tools such as the Hamilton Depression Rating Scale, devoid of the incorporation of objective biomarkers or neurobiological endpoints. Although certain RCTs indicate non-inferiority or comparable effectiveness to standard antidepressants like citalopram or fluoxetine, these outcomes have yet to be consistently reproduced in larger, multicenter investigations. Furthermore, assertions of superiority in some studies necessitate cautious interpretation due to the constrained sample sizes and the potential for bias. Notably, the majority of studies assess *Echium amoenum* primarily as a standalone treatment or in conjunction with pharmacological antidepressants, rather than within psychotherapeutic modalities such as CBT. Consequently, its prospective function as an adjunct to CBT remains conjectural and necessitates direct empirical exploration. Overall, while the current evidence suggests potential antidepressant properties of *Echium amoenum*, well-designed, large-scale, multicenter RCTs with standardized formulations and longer follow-up periods are necessary to confirm its clinical efficacy and mechanistic profile.

## 7 Mechanistic convergence between CBT and herbal interventions

CBT facilitates sustained clinical enhancement in MDD through mechanisms of cognitive restructuring, emotional regulation, and resilience to stress. Accumulating evidence from neuroimaging studies, molecular psychiatry, and psychoneuroimmunology indicates that CBT produces quantifiable biological effects, encompassing the modulation of neurotrophic signaling, alterations in monoaminergic transmission, and the regulation of inflammatory pathways. Notably, several of these molecular targets coincide with the mechanisms ascribed to *Lavandula angustifolia* and *Echium amoenum*, thereby substantiating their prospective function as adjunctive interventions that may biologically prepare patients for heightened responsiveness to CBT.

While the prospective synergistic interaction between CBT and the aforementioned herbal interventions is biologically conceivable, it is imperative to underscore that empirical clinical evidence endorsing this integrative approach remains scant. The majority of existing research has primarily focused on *Lavandula angustifolia* and *Echium amoenum* as standalone therapies or as adjuncts to pharmacological interventions, rather than in conjunction with psychotherapeutic modalities such as CBT. Consequently, the suggested synergism is predominantly predicated on intersecting neurobiological mechanisms, encompassing the modulation of neurotrophic signaling, monoaminergic pathways, and inflammatory processes, and must be approached with circumspection.

### 7.1 Shared molecular targets

One of the most significant domains of intersection between CBT and phytotherapeutic interventions resides in the modulation of BDNF and its downstream transcriptional modulator, cyclic adenosine monophosphate response element-binding protein (CREB). Empirical evidence indicates that CBT is capable of augmenting both peripheral and central levels of BDNF, particularly within the hippocampus and prefrontal cortex, which are regions essential for the regulation of emotions and executive functions (66, 67). The activation of CREB-mediated transcription facilitates synaptic plasticity, dendritic reorganization, and cognitive learning processes that form the foundation of cognitive restructuring within the context of psychotherapy. In a similar vein, bioactive constituents found within *Lavandula angustifolia* and *Echium amoenum* have been demonstrated to enhance BDNF expression and activate CREB signaling cascades in preclinical models addressing stress and depression (43, 60). These phenomena appear to be intricately associated with augmented monoaminergic neurotransmission, particularly in the realms of serotonergic and dopaminergic signaling, which are critical for the stabilization of mood, reward processing, and cognitive adaptability. Consequently, the integration of cognitive learning induced by CBT and molecular plasticity stimulated by herbal interventions may establish a conducive neurobiological milieu that promotes sustainable antidepressant outcomes.

### 7.2 Effects on neuroinflammation and oxidative stress

Neuroinflammation and oxidative stress play a crucial role in the pathophysiology of MDD by disrupting synaptic functionality, hindering neurogenesis, and modifying neurotransmitter metabolism. Increased concentrations of pro-inflammatory cytokines have been correlated with the severity of depressive symptoms and resistance to psychotherapeutic interventions. Importantly, CBT has been demonstrated to mitigate systemic inflammation and restore stress-related immune dysregulation, likely through the top-down modulation of the HPA axis and the autonomic nervous system (68, 69). Concurrently, *Lavandula angustifolia* and *Echium amoenum* exhibit pronounced anti-inflammatory and antioxidant properties. Lavender inhibits nuclear factor kappa B (NF-κB) signaling pathways and diminishes the production of pro-inflammatory cytokines, whereas *E. amoenum* effectively scavenges reactive oxygen species and bolsters endogenous antioxidant defenses, including superoxide dismutase and glutathione peroxidase (70, 71). By alleviating cognitive and emotional deficits induced by inflammation, these herbal agents may enhance patients’ abilities to engage in CBT-related tasks, such as attention, memory consolidation, and emotional processing.

From a translational neuroscience vantage point, the neurobiological mechanisms elucidated in this review, encompassing neuroinflammation and neuroplasticity, can be further elucidated within emergent systems-level paradigms that synthesize sleep regulation and cerebral clearance operations. Contemporary research indicates that the glymphatic system is paramount in the expulsion of metabolic byproducts from the central nervous system and may be functionally interconnected with both neuroinflammatory mechanisms and neuropsychiatric conditions. Disturbances in glymphatic clearance have been posited as an instrumental factor in the pathophysiology of mood disorders owing to compromised homeostatic management of neuroinflammation and neural plasticity. This integrative viewpoint may afford a more nuanced comprehension of how CBT and phytotherapeutic interventions might converge upon common biological systems that modulate cerebral function and emotional regulation (72, 73).

### 7.3 Implications for emotional regulation and cognitive restructuring

Effective emotional regulation and cognitive restructuring necessitate the preservation of prefrontal-limbic connectivity and adequate neuroplasticity. CBT enhances the top-down regulatory capacity of the prefrontal cortex over hyperresponsive limbic structures, such as the amygdala, leading to enhanced emotional regulation and diminished negative cognitive bias. These functional transformations are underpinned by molecular modifications, which include augmented BDNF signaling, mitigated inflammation, and stabilized monoaminergic activity. The supplementary application of *Lavandula angustifolia* and *Echium amoenum* may potentiate these mechanisms by attenuating affective reactivity, decreasing stress-induced arousal, and promoting neuroplastic readiness. Their anxiolytic, antidepressant, and anti-inflammatory properties may assist patients in attaining a neurobiological state that facilitates cognitive reappraisal and behavioral activation. Thus, the amalgamation of herbal interventions with CBT may constitute a personalized and mechanistically informed approach to enhance therapeutic outcomes in MDD, especially in individuals exhibiting elevated inflammation, stress sensitivity, or partial responses to psychotherapy alone.

### 7.4 Integrated molecular pathway model of *Lavandula angustifolia* and *Echium amoenum* in MDD

*Lavandula angustifolia* and *Echium amoenum* may manifest antidepressant properties through a convergent multi-target molecular network that encompasses neurotrophic, monoaminergic, and inflammatory mechanisms (74, 75). At the neurotrophic level, both interventions have been linked to the upregulation of BDNF and CREB signaling, which in turn fosters synaptic plasticity and hippocampal neurogenesis. At the monoaminergic level, the modulation of serotonergic and dopaminergic neurotransmission may facilitate enhanced mood regulation and cognitive flexibility. Concurrently, both agents demonstrate anti-inflammatory properties that are mediated by the inhibition of NF-κB signaling and the reduction of pro-inflammatory cytokines, such as IL-6 and tumor necrosis factor-alpha, thereby reinstating neuroimmune equilibrium. These converging pathways may ultimately mitigate hyperactivity of the HPA axis, bolster stress resilience, and promote neuroplastic adaptation, thereby establishing a biologically advantageous state that may augment responsiveness to CBT in individuals with MDD.

## 8 Potential synergistic effects of *Lavandula angustifolia* and *Echium amoenum* with CBT

The amalgamation of phytotherapeutic modalities with empirical psychotherapeutic paradigms has garnered heightened scholarly interest as a methodology to augment therapeutic effectiveness in individuals diagnosed with MDD. CBT predominantly addresses dysfunctional cognitive frameworks and emotional regulation through methodical educational interventions, whereas *Lavandula angustifolia* and *Echium amoenum* exert foundational biological influences on neurochemical, neurotrophic, and inflammatory pathways. The synthesis of these descending and ascending mechanisms offers a robust theoretical framework for synergistic interactions that may enhance clinical outcomes, diminish the likelihood of relapse, and fortify psychological resilience.

### 8.1 Neurobiological and psychological synergy

The synergy observed between CBT and herbal interventions arises from their synergistic impact on neural circuits associated with mood regulation, stress response, and cognitive control mechanisms. CBT promotes both functional and structural modifications within prefrontal–limbic networks, thereby enhancing the top-down modulation of amygdala-mediated emotional reactions and facilitating adaptive cognitive reappraisal processes. These neurobiological modifications are substantiated by an increase in neuroplasticity, particularly through the upregulation of BDNF and CREB signaling pathways, alongside the normalization of monoaminergic neurotransmission (66, 76). The botanicals *Lavandula angustifolia* and *Echium amoenum* may amplify these adaptations induced by CBT by biologically preparing the brain for enhanced learning and emotional adaptability. The anxiolytic and serotonergic properties of lavender serve to diminish autonomic hyperarousal and stress reactivity, thus enhancing attentional regulation and emotional involvement during CBT sessions (77, 78). Simultaneously, *E. amoenum* facilitates neurotrophic signaling and mitigates hyperactivity of the HPA axis, which may serve to alleviate stress-related deficits in memory consolidation and cognitive restructuring (79). From a psychological standpoint, the symptomatic alleviation afforded by these botanical agents, such as reductions in anxiety, sleep disruptions, and physical symptoms, may enhance motivation, adherence to treatment protocols, and self-efficacy, all of which are pivotal factors influencing the efficacy of CBT.

### 8.2 Effects on treatment response, relapse prevention, and resilience

The phenomenon of suboptimal treatment efficacy and elevated relapse frequencies continues to pose significant obstacles in the therapeutic management of MDD, even amongst individuals undergoing optimal psychotherapeutic interventions. The supplementary incorporation of *Lavandula angustifolia* and *Echium amoenum* alongside CBT may effectively target these vulnerabilities by engaging biological pathways that remain inadequately addressed by psychotherapy in isolation. Both of these botanical agents are characterized by their anti-inflammatory and antioxidant properties, which could potentially alleviate inflammation-mediated depressive manifestations that are linked to treatment resistance and the likelihood of relapse. Through the attenuation of pro-inflammatory cytokines and oxidative stressors, these herbal preparations may contribute to the preservation of synaptic integrity and the maintenance of neural adaptations induced by CBT over an extended period (80, 81). Furthermore, their potential to enhance BDNF-facilitated neurogenesis and synaptic remodeling may foster enduring emotional resilience and adaptability to stress. This comprehensive approach is congruent with personalized and preventative psychiatry, providing specific advantages for individuals exhibiting increased stress sensitivity, inflammatory biomarkers, or a partial response to conventional CBT. Despite the current scarcity of extensive RCTs, the available preclinical and clinical data substantiate the justification for forthcoming translational research investigating the synergistic effects of CBT and herbal interventions as a viable and low-risk augmentation strategy in MDD.

## 9 Clinical implications and translational perspectives

*Lavandula angustifolia* and *Echium amoenum* are presented in a variety of dosage forms that may enhance their practical application as adjunctive therapies alongside CBT for individuals diagnosed with MDD. *L. angustifolia* is frequently administered in the form of oral capsules, standardized extracts, teas, or aromatherapy formulations, with oral preparations being particularly advantageous for prolonged adjunctive treatment in depressive conditions. *E. amoenum* is predominantly ingested as aqueous extracts or infusions of dried flowers, although there is a growing interest in encapsulated standardized formulations to ensure consistency in dosing. The incorporation of *Lavandula angustifolia* and *Euphorbia amoenum* into contemporary clinical practice must be executed within a framework that is both evidence-based and centered around the patient. Their utilization as adjunctive therapies rather than substitutes for CBT is consistent with a multimodal treatment strategy that addresses both the psychological and neurobiological aspects of MDD.

## 10 Limitations, knowledge gaps, and future research directions

Notwithstanding the increasing scholarly attention directed towards *Lavandula angustifolia* and *Echium amoenum* as prospective antidepressant agents, the extant corpus of evidence is encumbered by numerous methodological shortcomings. A substantial number of preclinical investigations depend on acute or short-duration animal models of depression, which may inadequately reflect the chronic and heterogeneous characteristics of MDD. Furthermore, clinical investigations frequently encompass limited sample sizes, brief intervention periods, and diverse outcome measures, which constrains statistical power and the applicability of findings. Moreover, discrepancies in plant species, extraction methodologies, dosages, and administration routes further obfuscate interstudy comparisons and the reproducibility of results. A scant number of studies sufficiently account for placebo effects or evaluate long-term safety, and the neurobiological underpinnings of therapeutic response are seldom analyzed in conjunction with clinical outcomes. A significant deficiency in the existing body of knowledge pertains to the lack of rigorously structured RCTs that assess the cumulative effects of CBT in conjunction with phytotherapy. The majority of the extant clinical literature examines either herbal interventions or CBT in isolation, thereby offering limited understanding of their possible synergistic or additive effects. Subsequent RCTs ought to employ factorial or multimodal methodologies to facilitate direct comparisons between CBT administered independently, phytotherapy utilized independently, and interventions that combine both modalities. These investigations should evaluate not only the severity of depressive symptoms but also encompass cognitive, emotional, and functional outcomes, in addition to adherence to treatment and rates of relapse. Longitudinal study designs will be crucial in ascertaining the sustainability of effects and the preventive capabilities of these interventions. In order to augment translational relevance, subsequent investigations ought to employ biomarker-driven methodologies that correlate clinical outcomes with the fundamental molecular and neurobiological mechanisms. Research endeavors concentrating on neurotrophic factors, monoaminergic pathways, inflammatory cytokines, oxidative stress indicators, and neuroimaging correlates may elucidate the interactions between *L. angustifolia* and *E. amoenum* with the neuroplastic alterations induced by CBT. The incorporation of omics-based analyses alongside personalized medicine frameworks could further delineate patient subpopulations that are most likely to derive benefit from integrated interventions. Such mechanistic elucidations are pivotal for the optimization of treatment modalities and the progression of integrative strategies in MDD.

## 11 Conclusions

This comprehensive review elucidates the potential role of *Lavandula angustifolia* and *Echium amoenum* as complementary interventions to CBT in the treatment of MDD. An increasing body of evidence suggests that these phytotherapeutic agents demonstrate antidepressant and anxiolytic properties via converging neurobiological pathways, encompassing the modulation of monoaminergic neurotransmission, the enhancement of neurotrophic signaling, BDNF, as well as the attenuation of neuroinflammation and oxidative stress. These mechanisms partially overlap with the molecular and neuroplastic alterations engendered by CBT, thereby providing a biologically plausible—yet largely theoretical—framework for potential synergistic effects on mood regulation, cognitive processing, and emotional resilience. Importantly, it should be emphasized that current clinical evidence directly evaluating the combination of these herbal interventions with CBT remains limited, as most studies have investigated their effects as monotherapies or as adjuncts to pharmacological treatments. Therefore, any proposed synergistic benefit with CBT should be interpreted with caution. Anticipating future developments, integrative methodologies that synthesize psychotherapeutic modalities with empirically validated phytotherapeutic interventions may represent a promising direction in individualized and preventive mental health care. Such multimodal strategies have the potential to enhance treatment efficacy, mitigate symptom recurrence, and accommodate interindividual variability in therapeutic outcomes. However, contemporary clinical investigations indicate that the efficacious antidepressant properties of *Lavandula angustifolia* are predominantly noted in standardized oral preparations such as Silexan (typically administered at a dosage of 80 mg/day) or analogous essential oil-based formulations, whereas *Echium amoenum* has been predominantly examined in aqueous or hydroalcoholic extracts within a dosage range of approximately 375 mg/day, albeit no universally recognized optimal dosage has been established to date. The existing evidence concerning dose-response relationships remains limited and heterogeneous across various studies, thereby obstructing the formulation of definitive dosing recommendations, whether utilized as monotherapy or as adjunctive therapy. Furthermore, the data pertaining to long-term safety and efficacy for both *Lavandula angustifolia* and *Echium amoenum* are presently inadequate. The majority of clinical trials have been of a short-term nature (spanning 4–8 weeks), and there exists a deficiency of substantial evidence addressing sustained usage, relapse prevention, or the safety profiles associated with chronic administration. Consequently, longitudinal follow-up studies and post-marketing surveillance are imperative to delineate long-term risk-benefit ratios. Nonetheless, the successful application in clinical settings will rely on the execution of rigorous RCTs, particularly those specifically designed to assess combined interventions with CBT, the establishment of standardized herbal formulations, and the implementation of biomarker-driven patient stratification. Furthermore, the existing evidence base is limited by factors such as small sample sizes, short follow-up durations, and heterogeneity in study design, which should be addressed in future research. Ongoing interdisciplinary inquiry that connects neuroscience, psychiatry, and herbal medicine is imperative to ascertain safety, efficacy, and mechanistic understanding, thereby ultimately facilitating the judicious integration of complementary therapies into comprehensive treatment frameworks for MDD.
